# Current practices, gaps, and opportunities on the role of clinical pharmacists in cancer pain management: Perspectives from Nepal

**DOI:** 10.1177/10781552231205025

**Published:** 2023-10-17

**Authors:** Sunil Shrestha, Siew Hua Gan, Vibhu Paudyal, Bhuvan KC, Simit Sapkota

**Affiliations:** 1 School of Pharmacy, 65210 Monash University Malaysia, Bandar Sunway, Selangor, Malaysia; 2 School of Pharmacy, College of Medical and Dental Sciences, Sir Robert Aitken Institute for Medical Research, 1724 University of Birmingham Edgbaston, Birmingham, UK; 3 School of Clinical Sciences, 1969 Queensland University of Technology, Brisbane, Australia; 4 Department of Clinical Oncology, 608992 Kathmandu Cancer Center, Tathali, Bhaktapur, Bagmati Province, Nepal; 5 Department of Clinical Oncology, 533641 Civil Service Hospital, Minbhawan, Kathmandu, Bagmati Province, Nepal

**Keywords:** Cancer pain, low-middle-income countries, medication review, pharmacist, Nepal

## Abstract

N/A

## Background

Cancer continues to be a significant global health issue, with a projected increase in incidence in the coming years.^1^ According to GLOBOCAN, an estimated 19.3 million new cancer cases were diagnosed in 2020, accounting for 9.9 million deaths, a rise from previous years.^2^ In fact, approximately half of all cancer cases and more than half of all cancer deaths are thought to occur in Asia.^2,3^ In 2018, according to GLOBOCAN, there were 26,184 new cancer cases and 19,413 cancer deaths in Nepal alone.^4^ The age-adjusted incidence rates for cancer were 95.3 (men) and 98.1 (women) among the 3 million people living in the Kathmandu Valley, with corresponding death rates of 36.3 and 27/100,000, respectively.^5^

Nepal is a low-resource country in Asia, situated between China and India. It is a developing nation that recognizes healthcare as a fundamental right for its citizens.^6^ Nevertheless, the country faces challenges in providing adequate health services due to having limited resources and infrastructure. Therefore, cancer remains a significant public health challenge in Nepal, contributing to 10% of its overall mortality rate.^7^ The observed contribution of cancer to Nepal's overall mortality rate, comprising 10% of total deaths,^7^ requires a nuanced examination of the global and low-middle-income country (LMIC) benchmarks. The significance of this proportion must be considered within the broader context of international mortality patterns, accounting for variables such as demographic characteristics, healthcare infrastructure, prevalent risk factors, disease prevalence, and vital records keeping. Nepal's cancer-related mortality rate is 10%, a significant difference compared to high-income countries such as the US or the UK. However, it's essential to recognize that LMIC data can vary due to factors like healthcare accessibility and resources. While this comparison provides insight, it's essential to consider the unique healthcare context of Nepal. This emphasizes the importance of cross-country studies for a comprehensive understanding of the contribution of Nepal to global cancer-related mortality. The cancer burden in Nepal is substantial, with rising cancer incidence and mortality rates contributing significantly to the disease burden measured in disability-adjusted life years (DALYs). The most prevalent types of cancer in Nepal include breast cancer, lung, cervical, stomach, and oral cavity cancers.^7^ The limited availability and accessibility of cancer care services in Nepal, particularly in rural areas, further compound this burden.^7–9^ Given the high disease burden, Nepal urgently requires reinforced efforts to strengthen cancer prevention, early detection, diagnosis, treatment, pain management, and palliative care. Such steps encompass raising public awareness of cancer risk factors, advocating healthy lifestyle behaviors, enhancing cancer screening and diagnostic services, and expanding access to vital cancer treatment modalities such as chemotherapy, radiotherapy, surgery, and potentially immunotherapy.^9–11^

Pain is a severe and distressing cancer symptom that impacts patients’ quality of life (QoL). Despite its profound implications, Nepal lacks comprehensive epidemiological data on the burden of cancer pain. Although pain management is an indispensable pillar of effective cancer care, the research setting has yet to provide a holistic understanding of this critical aspect. While palliative care encompasses a broader spectrum of supportive interventions, including pain management, it is essential to distinguish between the two. To date, limited research has shed light on the pressing need for comprehensive palliative care strategies in Nepal, highlighting the multifaceted challenges faced in addressing pain and other aspects of end-of-life care.^12–15^ In the current scenario, accessibility to palliative care and essential pain management medications, such as opioids, remains a formidable challenge, particularly in rural areas where the need is often most acute.^13,14^ Recognizing the urgency of this issue, the government of Nepal has taken significant steps towards addressing this gap. Initiatives such as including palliative care in the national health policy and adopting a dedicated national palliative care strategy underscore the commitment of Nepal to ensuring equitable access to comprehensive palliative care services for all its citizens, irrespective of geographical location.^16^

Nepal faces significant challenges in providing palliative care due to limited public and private services and a lack of recognition of palliative care as a specialized field.^17^ The situation is compounded when, to date, the recognition of the role of palliative care as a specialized field has been limited.^18^ To address this, there's a need to develop para-professional palliative care specialists, optimize information technology tools, and reform the care delivery system.^18^ The Nepalese Association for Palliative Care offers resources through homepalliativecarenepal.net, aligning with the 2014 World Health Assembly Resolution WHA67.19 to strengthen palliative care.^19^ The Nepal Society for Palliative Care has a ten-year vision for comprehensive, accessible, and affordable palliative care across various healthcare settings, focusing on education, training, drug availability, reporting, and research. Approximately 62,000 individuals require palliative care annually in Nepal, primarily for cardiovascular disease, cancer, and chronic pulmonary disease.^20^

In the 2017 edition of the Worldwide Hospice Palliative Care Alliance (WHPCA)/WHO Global Atlas of Palliative Care, Nepal was classified under Group 3a.^21^ This category encompasses nations with fragmented palliative care provision, characterized by a limited scope of palliative care activism, dependency on donor funding, restricted access to morphine, and a relatively small number of palliative care services compared to the population size.^21^ Some cancer hospitals in Nepal, including B.P. Koirala Memorial Cancer Hospital, Bhaktapur Cancer Hospital, Nepal Cancer Hospital and Research Center, Kathmandu Cancer Center, Kanti Children's Hospital (KCH), Green Pastures Hospital (Pokhara), Bir Hospital, Civil Service Hospital, Patan Hospital, National Cancer Hospital, Manipal Medical College, Sushil Koirala Cancer Hospital in Nepalgunj, Purbanchal Cancer Center, BPKIHS Dharan, and independent hospices like Hospice Nepal and Thankot Hospice, offer palliative care services. However, most of the hospitals lack palliative care specialists.

## Cancer pain management in Nepal

Cancer pain is pervasive in Nepal, affecting both patients and their families. Despite efforts by the Nepalese government to improve access to pain management services and train medical professionals in pain management techniques, the current standard of cancer pain management in Nepal remains below par. In fact, many cancer patients experience uncontrolled pain and suffering due to several critical factors listed below:

1. *Lack of proper medical facilities:* The inadequate availability of specialized medical facilities for cancer pain management poses a significant challenge.^9,10^ The absence of dedicated pain management clinics and centers contributes to suboptimal pain control for cancer patients.
2. *Limited availability of opioid medications:* Despite attempts by the government to increase the availability of opioid medications, some challenges remain in ensuring a consistent and sufficient supply of these essential drugs.^22^ Opioids are a cornerstone of effective cancer pain management, and their scarcity hampers optimal pain control for patients.^23,24^
3. *Awareness and training gap:* While training programs have been introduced, there is a notable gap in awareness and training among medical professionals in managing cancer pain using opioids.^10^ The lack of understanding and expertise contributes to the underutilization of opioid medications and suboptimal pain relief.
4. *Shortage of trained medical professionals:* The shortage of trained medical professionals, particularly those specializing in palliative care and pain management, contributes to the inadequate pain management experienced by cancer patients.^10^ The burden of managing cancer pain often falls on oncologists who may not have specialized training in pain management.
5. *Lack of availability of pain management close to home*: Due to lack of availability close to the patient's home, many patients must travel a long distance to get medicine, which becomes challenging and more complex as the disease progresses. Consequently, medicine breaks and inadequate treatment are quite common. The limited availability of home care facilities further compounded the problem.

Pain is a prevalent symptom among cancer patients an estimation of 66% of patients reported having experienced pain.^25^ Experiencing pain can significantly impact a patient's health-related QoL and increase healthcare utilization and expenditures.^26,27^ Cancer-related pain can also affect the physical, psychological, and social functioning of the patients.^28,29^ To improve cancer pain management in Nepal, the Nepalese government has introduced various initiatives, including palliative care programs, the establishment of pain management clinics, and training programs for medical professionals. However, the success and effectiveness of these initiatives are complex and multifaceted, requiring further investigation and data to ascertain their impact.

Despite various efforts to improve cancer care services in Nepal, a significant challenge remains, including the limited availability of resources, particularly in rural areas where most of the population also resides.^20^ Therefore, various aspects of healthcare, such as physical infrastructure, human resources, and training programs, must be enhanced.^9^ Access to effective pain management for cancer patients is compromised due to inadequate healthcare infrastructure and resources.^20^ Addressing these challenges demands substantial investment in healthcare infrastructure, training medical professionals in pain management, and ensuring the availability of essential pain management drugs. The role of palliative care specialists in Nepal remains limited, with oncologists primarily managing cancer pain within busy clinics where palliative treatment may be overlooked.

Although efforts to improve public awareness of cancer pain management and enhance accessibility to services are gradually being introduced by the government, significant gaps exist. While private facilities can offer some alternatives, they remain financially out of reach for many Nepalese patients. The reliance on seeking cancer treatment in neighboring India highlights the need for comprehensive follow-up care options within the country itself.

Through increased resource allocation and targeted interventions, there is potential to improve the QoL of cancer patients and their families. Enhancing cancer pain management in Nepal is essential for the well-being of an individual patient and is crucial for reducing the country's overall burden of cancer pain. Continued efforts and investments promise to make a meaningful difference in the lives of those affected by cancer-related pain in Nepal.

## Clinical pharmacy practice in Nepal

Clinical pharmacy services hold a pivotal role in cancer pain management,^30,31^ especially within the context of Nepal's healthcare landscape. These services are essential in ensuring that cancer patients receive optimal pain management, a critical component of their overall care.^31^ Pharmacists can ensure safe medication use, dispense drugs, counsel patients, and monitor for side effects. Their roles have evolved to include more comprehensive care and collaboration with other providers, making them crucial in a patient-centered approach.^31,32^ Since drug management is becoming more complex, pharmacists play even more significant roles in improving outcomes while reducing costs.^31^ The ability of pharmacists to effectively manage cancer pain is due to their specialized training in medication and clear understanding of drug–drug interactions (DDIs) and adverse drug reactions (ADRs). The role becomes more important since most cancer patients have multiple co-morbidities and are on polypharmacies.^33,34^ Although DDIs and ADRs should be paramount when prescribing pain medications, these factors are often overlooked in clinical practice. When there is a lack of dedicated pain management personnel or sufficient support, clinical pharmacy services need to step in to provide personalized interventions and collaborate seamlessly with other healthcare providers. In this way, the successful implementation of pain management strategies facilitated by clinical pharmacy services helps alleviate the adverse impact of debilitating pain on the QoL experienced by cancer patients.

Clinical pharmacy practice is a multifaceted field encompassing various healthcare settings, including inpatient units and specialized areas such as cancer practice, pain management, and palliative care. While our work primarily focuses on integrating clinical pharmacy services within the broader healthcare context, it is essential to note that the specific areas of cancer practice, pain management, and palliative care are integral components of this practice. Although clinical pharmacy services have been established in many hospitals in Nepal, the number of clinical pharmacists providing these services remains low, and there is a lack of institutions and programs offering practice-focused clinical pharmacy training.^35^ Furthermore, while most hospitals possess pharmacy services, these services predominantly revolve around procurement, distribution, and inventory management rather than encompassing the essential core clinical pharmacy functions.^35^ The Ministry of Health and Population in Nepal established a guideline in 2015 that outlined the optimal number of clinical pharmacists required to provide clinical pharmacy services to hospitals with varying bed capacities.^36^ Specifically, the guideline recommended that hospitals with 51–100 beds have at least one clinical pharmacist available. Unfortunately, despite developing the said guideline,^36^ its implementation remains a challenge in government hospitals of Nepal.

Existing literature^35,37–39^ reflects the challenges faced in clinical pharmacy services. Among the factors, limited career prospects, inadequate remuneration, and barriers related to pharmacy education have been reported to impede the growth of this field. Moreover, clinical pharmacy services encounter obstacles, including poor public awareness, communication issues among pharmacists, uncertainties in roles and responsibilities, and a lack of recognition and acceptance by other healthcare professionals.^37^

The attitudes and perceptions of medical professionals play a crucial role in influencing the integration of clinical pharmacy services. Furthermore, disparities in acknowledgment can result in hesitancy toward collaboration. This is particularly evident in private tertiary care hospitals where qualified PharmD and M. Pharm graduates are sometimes underutilized due to perceived value gaps, thus impeding their engagement as clinical pharmacists.

To address these challenges, efforts to raise awareness among healthcare professionals and the public about the value of clinical pharmacy services are crucial. Targeted training, partnerships with pharmacy professionals, and increased engagement by medical professionals can bridge the recognition gap and enhance integration.^37^ Moreover, educating pharmacy students with practical skills through robust internships and hands-on education can bolster their competency in actual practice.^37^

## Key aspects of clinical pharmacy services in cancer pain management

Successful cancer pain management necessitates a comprehensive, multidisciplinary approach that covers crucial facets of patient care. Clinical pharmacy services substantially enhance the quality of cancer pain management. They offer specialized proficiency in medication management, monitoring ADRs, patient education, and beyond. Table 1 highlights pivotal dimensions of clinical pharmacy services that augment cancer pain management. Each dimension emphasizes the vital role of clinical pharmacists in optimizing pain control, elevating patient outcomes, and ultimately enhancing the QoL for individuals who have cancer.

**Table 1.** Aspects of clinical pharmacy services.

Aspects of clinical pharmacy services	Description
**Patient history**	A patient history is crucial to identify pain triggers, intensity, frequency, and response to previous treatments.^40^ Clinical pharmacists need a comprehensive patient history to create a tailored and safe pain relief plan that aligns with the individual's requirements. When gathering a patient's history, factors like pain treatment preferences, pain history, pain description, sociodemographic details, cancer-related information, vocal, or facial cues from the patient, past treatment experiences, average pain rating over the past week, current pain rating, and treatment adherence history should all be considered.
**Medication review and dosing adjustments**	Medication review is one of the imperative tools for pharmaceutical care, and it is essential to ensure that cancer patients receive the correct medications and dosages for effective pain management.^41^ Clinical pharmacists monitor medication regimens and adjust the doses to help patients manage pain and achieve optimal health outcomes.^42^ They can also assist in identifying and managing drug interactions and adverse effects.^42^
**Adverse drug reactions (ADRs) monitoring**	Another vital aspect of clinical pharmacy service is ADRs monitoring. Cancer treatments, including pain medications (e.g. opioids), can cause adverse effects (e.g. constipation, sedation, dizziness, nausea, vomiting, physical dependence, tolerance, and respiratory depression), and clinical pharmacists can help monitor these effects while educating patients on how to report them should they occur.^43,44^ Also, clinical pharmacists can help manage and prevent the impact in the future.
**Drug interactions**	Drug–drug interactions (DDIs) are a primary concern for cancer patients. DDIs can contribute to opioid overuse, misuse, abuse, morbidity, and mortality.^45^ Clinical pharmacists can help identify potential drug interactions and minimize interaction risks by working with other healthcare team members.^46^
**Physical examination**	Physical examination is crucial to cancer pain management and provides valuable information. Pharmacists can assess a patient's posture, movement, and gait and be on the lookout for signs of pain or discomfort,^47^ like grimacing or eye closing.
**Pain scale assessment**	Assessing pain using a pain scale [e.g., Numerical Rating Scale (NRS) of 0 (no pain) to 10 (worst pain imaginable), categorical scale or visual analog scale (no pain, mild pain, moderate pain, or severe pain, Brief pain inventory (BPI)] enables determining appropriate analgesic dosage. Pharmacists play a crucial role in actively listening to patients and gathering information to design a good analgesic medication regimen.^48^
**Patient education and counseling**	Patient education and counseling are crucial to comprehensive cancer pain management.^12,48^ By educating patients on their condition, available treatments, and expected outcomes, pharmacists can help dispel misconceptions and alleviate anxiety and fear while increasing adherence to prescribed analgesic regimens.^49^ Pharmacists can also prepare educational materials (e.g. leaflets) for patients and their family caregivers, providing psychosocial support and education.^49^
**Support to the team**	The pharmacist can assist medical oncologists or treating physicians in determining the most effective treatment options based on the patient's response to previous treatments, potential contraindications, and underlying medical conditions. The pharmacist can also assist other health professionals such as nurses.

Clinical pharmacy services are vital for a comprehensive and holistic approach to managing cancer pain. Clinical pharmacists play a pivotal role by conducting thorough patient assessments, reviewing medications, and offering patient education. They address pain triggers, optimize medication plans, avert ADRs, and pinpoint potential interactions. Through collaborative efforts within multidisciplinary teams, clinical pharmacists are instrumental in reducing suffering and improving the well-being of cancer patients. This marks a significant stride in comprehensive cancer care.

## Opportunities and challenges for LMICs

A recent systematic review and meta-analysis conducted by Shrestha et al.^31^ have shed light on the critical need for further exploration of the role of pharmacists in cancer pain management, particularly within LMICs. The body of research in this area predominantly emanates from high-income countries like the United States, Canada, Japan, and China, where pharmacists have demonstrated involvement in diverse services, including medication reviews, patient counseling, ADR detection, and pain assessment either independently or in collaboration with other healthcare providers, LMICs remain a less explored territory.^31^ The study highlights that pharmacist interventions, encompassing pharmacological and non-pharmacological methods, have effectively improved clinical outcomes. These interventions lead to reduced pain intensity, mitigation of ADRs, and increased patient satisfaction across diverse settings in high-income countries.^31^ As such, it is imperative to extend our focus towards developing the roles of pharmacists in LMICs, addressing the existing gaps in knowledge concerning cancer pain management, and identifying potential barriers to pharmacist engagement.

The existing data emphasize pharmacist interventions in enhancing clinical outcomes and patient satisfaction in high-income countries.^31^ Given the distinctive obstacles encountered in low-resource nations, there is a need to investigate flexible and economic approaches to incorporate pharmacist interventions into cancer pain management. In LMICs, where restricted medication access is a prominent issue, non-pharmacological strategies like patient education and counseling may be exceptionally valuable. Additionally, involving clinical pharmacists in ward rounds can yield pragmatic advantages and cost savings.^31^ Moreover, empowering and training healthcare personnel, including pharmacists, can offer a potent solution to addressing the scarcity of skilled individuals in pain management within such contexts. Consequently, additional research is needed to explore how pharmacist-led interventions can be adapted to overcome resource limitations in LMICs. Such studies hold the potential to uncover interventions suitable for adoption in low-income settings, providing valuable insights to enhance cancer pain management strategies and ultimately improve the QoL for individuals dealing with cancer.

## How can clinical pharmacy services improve the quality of cancer pain management?

1. Improving cancer patient care with clinical pharmacy services requires a focused, strategic approach. Guided by precise recommendations, the goal is to bridge gaps and optimize outcomes. To successfully integrate clinical pharmacy services into cancer pain management in LMICs, transitioning from identifying care aspects to actionable improvement steps is crucial. Specialized Training and Capacity Building: Focused education and training programs for clinical pharmacists should be developed to equip them with the necessary skills to excel in cancer pain management. Training modules can cover pain assessment techniques, patient counseling strategies, monitoring ADRs, and drug interaction interventions.
2. *Collaborative multidisciplinary care:* Integrating clinical pharmacists into multidisciplinary oncology teams fosters seamless collaboration with other healthcare professionals.^50–52^ The integration ensures a holistic patient-centered approach, where pharmacists contribute their expertise in medication management to enhance overall patient outcomes.
3. *Protocol development and standardization:* Developing standardized protocols and guidelines that accommodate the unique challenges and opportunities of LMICs for cancer pain management, endorsed by medical associations and healthcare institutions, can provide a framework for consistent and evidence-based practice.^53^ The protocols should encompass pain assessment tools, dosing guidelines, medication selection criteria, and patient education materials.^54^
4. *Pharmacist prescribing authority:* Exploring the expansion of pharmacist prescribing authority, as seen in countries like the UK, can enhance the efficiency of cancer pain management.^55^ In fact, enabling pharmacists to prescribe pain medications independently empowers them to make real-time decisions, improving patient access to timely and appropriate care.
5. *Technology integration:* Implementing technology solutions, such as electronic health records and telehealth platforms, can facilitate remote consultations and medication management,^56,57^ particularly for patients in remote or underserved areas.
6. Quantitative staffing targets or establishing a defined ratio of clinical pharmacists to patient beds can help ensure comprehensive coverage and availability of their services. Drawing from international best practices and tailoring them to the local context can provide a standardized approach while accounting for variations in patient load and resource constraints.
7. *Research and outcome measurement:* Rigorous research studies are needed to quantify the impact of clinical pharmacy services on cancer pain management within LMICs. This includes assessing pain intensity, pain relief, patient outcomes, QoL improvements, medication-related adverse events, DDIs, and healthcare cost savings. Additionally, knowledge related to pain management and patient satisfaction with pain management services can be assessed.

It is essential to tailor the implementation approach to the specific healthcare landscape of each LMIC, considering factors such as existing infrastructure, available resources, and cultural considerations. A stepwise approach that starts with targeted pilot projects and gradually expands to broader implementation can ensure feasibility and sustainability.

## Recommendations

To unlock the full potential of clinical pharmacy services in the realm of cancer pain management, it is imperative to consider the following vital recommendations for a comprehensive and patient-centric approach

1. *Strategic expansion:* Systematically expand the integration of clinical pharmacy services across hospitals and healthcare institutions, ensuring adequate coverage and accessibility for cancer patients.
2. *Collaborative partnerships:* Foster robust collaborations between clinical pharmacists and the broader healthcare team, emphasizing a multidisciplinary approach to cancer pain management.
3. *Specialized training:* Develop tailored training programs that equip clinical pharmacists with specialized skills in pain assessment, medication review, medication management, morphine dose calculation, patient education, and counseling.
4. *Evidence-based practice:* Encourage developing and adopting standardized protocols and guidelines for cancer pain management grounded in the latest evidence.
5. *Advocacy and awareness:* Promote public and healthcare professional awareness regarding the instrumental role of clinical pharmacy services in enhancing cancer pain management and patient outcomes.

## Conclusion

This commentary underscores the pivotal role of clinical pharmacy services in elevating cancer pain management and overall patient outcomes within healthcare framework of Nepal. While dedicated cancer hospitals play a crucial role, and practice variations highlight the imperative to bridge current gaps. Clinical pharmacists contribute significantly to a holistic care strategy with their expertise spanning medication management, dosing adjustments, patient education, ADRs prevention, and drug interaction monitoring. Beyond medication dispensation, their collaborative synergy with medical oncologists, physicians, nurses, and fellow healthcare professionals enhances pain management and overall care quality. The proposed recommendations, encompassing strategic expansion, collaborative partnerships, specialized training, evidence-based practice, and advocacy initiatives, collectively represent vital steps toward realizing the full potential of clinical pharmacy services in Nepal, not only addressing existing challenges in comprehensive cancer care but also ensuring that cancer patients access highly trained professionals for a comprehensive approach to pain management, ultimately improving their QoL.
